# Developmental features and predicting airway failure risk in critically ill children with mandibular hypoplasia using 3D computational tomographic analysis

**DOI:** 10.1038/s41598-021-89302-4

**Published:** 2021-05-10

**Authors:** Doo-Hwan Kim, Eunseo Gwon, Junheok Ock, Jong-Woo Choi, Jee Ho Lee, Sung-Hoon Kim, Namkug Kim

**Affiliations:** 1 Department of Anesthesiology and Pain Medicine, Asan Medical Center, University of Ulsan College of Medicine, Olympic-ro 43-gil, Songpa-gu, Seoul, 138-736 Korea; 2 Department of Convergence Medicine, Asan Medical Center, University of Ulsan College of Medicine, Seoul, Korea; 3 Department of Plastic Surgery, Asan Medical Center, University of Ulsan College of Medicine, Seoul, Korea; 4 Department of Oral and Maxillofacial Surgery, Asan Medical Center, University of Ulsan College of Medicine, Seoul, Korea

**Keywords:** Risk factors, Oral anatomy, Medical imaging

## Abstract

In children with mandibular hypoplasia, airway management is challenging. However, detailed cephalometric assessment data for this population are sparse. The aim of this study was to find risk factors for predicting difficult airways in children with mandibular hypoplasia, and compare upper airway anatomical differences using three-dimensional computed tomography (3D CT) between children with mandibular hypoplasia and demographically matched healthy controls. There were significant discrepancies in relative tongue position (*P* < 0.01) and anterior distance of the hyoid bone (*P* < 0.01) between patients with mandibular hypoplasia and healthy controls. All mandibular measures were significantly different between the two groups, except for the height of the ramus of the mandible. After adjusting for age and sex, the anterior distance of hyoid bone and inferior pogonial angle were significantly associated with a difficult airway (*P* = 0.01 and *P* = 0.02). Quantitative analysis of upper airway structures revealed significant discrepancies, including relative tongue position, hyoid distance, and mandible measures between patients with mandibular hypoplasia and healthy controls. The anterior distance of the hyoid bone and inferior pogonial angle may be risk factors for a difficult airway in patients with mandibular hypoplasia.

## Introduction

In patients with craniofacial developmental deformity such as mandibular hypoplasia, airway management is critical for patient safety but remains challenging^1^. These patients are generally encountered in the intensive care unit or operating room due to various co-morbidities^2^. Failure to predict a difficult airway during anesthesia or critical care practice can cause severe morbidity and mortality^3^. Thus, reliable tools for precise diagnosis and prediction of difficult airways before practice are important. However, detailed configurational assessment data for these populations is sparse, and actionable airway securing strategies remain anecdotal.

Adequate preoperative airway planning, including patient-specific techniques and equipment, can decrease the risk associated with difficult airway management^4^. There is considerable demand for a valid tool to evaluate airway patency and predict difficult intubation in patients undergoing surgery or receiving optimal care. Multifactorial assessments using individual airway tests and difficult airway risk factors may help preoperative airway planning and difficult airway prediction^5,6^. However, recently, a large database study using multivariable assessments could not improve difficult airway prediction^7^. Although radiologic assessments, such as cephalometry, can provide valuable skeletal information on upper airway patency^8^, it provides only two-dimensional representations of three-dimensional (3D) structures, which cannot provide volumetric data or evaluate soft tissue structures. A 3D digitalized quantitative measurement of anatomical structures can overcome these limitations of traditional cephalometric analysis and provide useful information for airway management^9,10^.

This study aims to quantitatively analyze structural variations of the mandible, tongue, and airway, as well as their growth retardation patterns in children with mandibular hypoplasia compared to normal controls. The objective was to better understand the early natural history of mandibular hypoplasia and to find clinically useful risk factors for predicting difficult airways.

## Methods

### Patient selection

This study protocol was approved by our Institutional Review Board of the Asan Medical Center (Approval Number 2018-0967). From January 1990 to June 2018, patients diagnosed with mandibular hypoplasia, including Pierre-Robin sequence (PRS), Treacher-Collins syndrome (TCS), Goldenhar’s syndrome (GS), and hemifacial microsomia (HM)^2^, were included in this study if they had an available facial bone or head computed tomographic (CT) scan. We excluded patients with incomplete documentation of clinical and demographic data. To ensure we meet all image quality criteria, we excluded patients with the following conditions; (1) accurate parameters could not be obtained because of artifacts on the CT scan, (2) landmarks to reconstruct 3D multi-planar reformatting and to measure the upper airway were not included in the CT scans.

Healthy controls were identified from existing CT data in our institution’s picture archiving and communication system. Healthy control participants were age, sex, height, and weight-matched to those with mandibular hypoplasia. The morphological growth pattern of the mandible is strongly related to the dentition development stages^11^; hence, subgroup analyses were conducted according to the stage of dentition (≤ 5 and > 5 year-old group)^12^.

### Measurements and data collection

**Figure 1:**
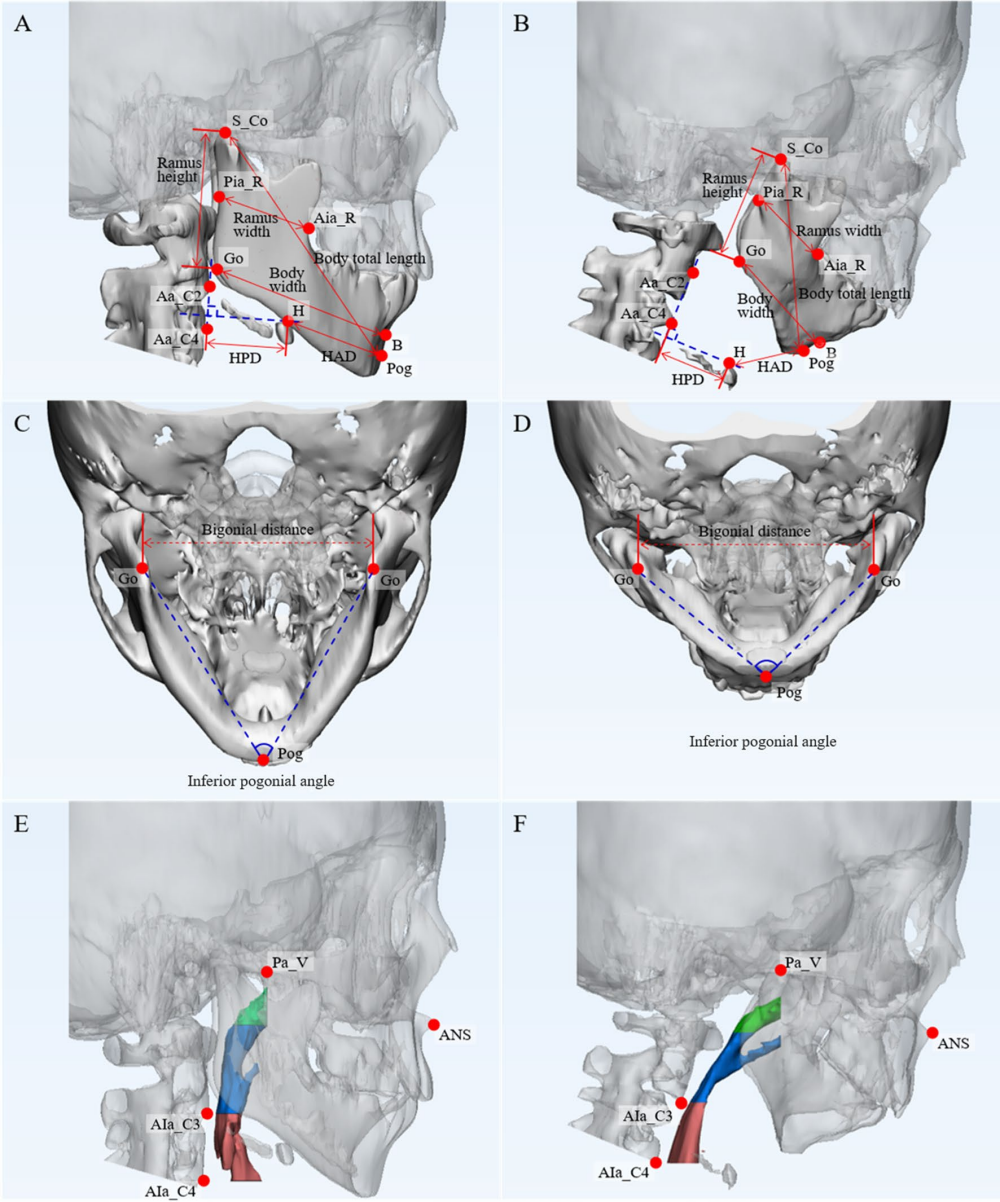
(**A**) Main variables of mandible measurement in the ramus, mandibular body, and position of the hyoid bone in healthy controls (**A**) and patients with mandibular hypoplasia (**B**). *S_Co* Superior condylion, *Pia* Posterior inner aspect of the ramus, *Aia* Anterior inner aspect of the ramus, *Go* Gonion, *Pog* Pogonion, *Aa_C2* Anterior aspect of C2, *Aa_C3* Anterior aspect of C3, *HAD* Hyoid anterior distance, *HPD* Hyoid posterior distance. Bigonial distance and inferior pogonial angle in healthy controls (**C**) and patients with mandibular hypoplasia (**D**). Upper airway volumes in healthy controls (**E**) and patients with mandibular hypoplasia (**F**). Green, blue, and red highlights indicate the nasopharynx, oropharynx, and hypopharynx, respectively. *Pa_V* Posterior aspect of vomer, *ANS* Anterior nasal spine, *AIa_C2* Anterior inferior aspect of C2, *AIa_C4* Anterior inferior aspect of C4.

To analyze the computed tomography scan images, Mimics and 3-matic (Materialise, Leuven, Belgium) software were used. First, each scan was re-sliced to align the head position and clarify the skull alignment. The upper airways of patients with mandibular hypoplasia were analyzed according to the 3D multi-planar reformatting (MPR) plane. The average Frankfort horizontal plane (AFH_p) was defined using four points: Orbitale left, Orbitale right, Porion left, and Porion right. To form an average plane using the four points, the following process was required; (1) take the four landmarks required to define the AFH_p; (2) exclude each of the four points one-by-one and generate a total of the four planes using three points; (3) at (2) to generate an AFH_p using the average of existing planes algorithm of the 3-matic from the four planes. 3D MPRs were reconstructed using the midsagittal plane that passed through two points (Nasion, Opisthion) perpendicular to the average FH plane, and the coronal vormer plane that passing through the posterior aspect of the vomer in the coronal direction—reoriented at the average FH plane and midsagittal plane—and AFH_p. The hard and soft tissue landmarks needed to measure are defined in the reconstructed 3D MPR view in Supplementary Table S1 online. A total of 30 defined landmarks were included in the cephalometric analysis to measure upper airways. Two experts independently measured 30 landmarks of 84 CT scan datasets for upper airway analysis. The surface area, volume, distance and angle between particular landmarks were measured in children with mandibular hypoplasia and healthy controls (Fig. 1), according to the definition stipulated in Supplementary Tables S1 and S2 online.

Collected demographics included age, weight, height at the time of CT scan, sex, and patients’ diagnoses. Perioperative variables such as history of surgery, tracheostomy, difficult airway, and type of airway maintenance device were retrieved from the electronic medical record. Based on the practice guideline^13^, the ‘Difficult airway’ was defined as a clinical situation in which experienced attending staff anesthesiologists (> 10 years) encountered difficulty with facemask ventilation or difficulty with tracheal intubation. Difficult mask ventilation refers to mask ventilation that is inadequate, unstable, or requires two providers with or without a muscle relaxant^14^. Difficult tracheal intubation means that tracheal intubation requires multiple attempts, in the presence or absence of tracheal pathology^13^.

### Statistical analysis

Statistical analysis was performed using the R software version 3.5. Data are presented as mean ± standard difference (SD) or median [interquartile range (IQR)]. Student's t-test or the Mann–Whitney U test was used to compare the groups, as appropriate. The inter-rater reliability of the two observers was analyzed using MedCalc Software (Mariakerke, Belgium). To determine the association between upper airway measurements and age, we performed a linear regression with logarithmic transformations on each patient with mandibular hypoplasia and each healthy control. Then, we calculated the logistic regression equation of each parameter between the two groups. Based on the obtained logistic regression equation of the healthy controls, differences (∆) between estimated values according to the regression equation and measurement value of each parameter were then determined. To investigate the risk factors for a difficult airway, the area under the receiver-operating characteristic (ROC) curve of ∆ parameter was generated. As age and sex could play an important role in mandibular development^11^, logistic regression, adjusted for age and sex, was used to assess the association between upper airway measurements and difficult airway status. A *P* < 0.05 was considered statistically significant.

To determine inter-observer variability, 18 of the 30 landmarks in 40 CT scan data were measured. Using a total of 720 landmarks, inter-rater reliability was calculated using the Bland–Altman analysis on the distance between each landmark and the origin and mean and SD of the distance between the same landmarks by observer 1 and 2.

### Ethics approval

This study was performed according to the Declaration of Helsinki. The current study protocol was approved by the institutional review board of Asan Medical Center, Seoul, Korea (Approval Number: 2018-0967). Due to the retrospective nature of the study, informed consent was waived.

## Results

In total, 70 patients with mandibular hypoplasia were included. Twenty-one patients with incomplete documentation of clinical data and seven patients without an adequate quantitative CT scan were excluded. Forty-two patients with congenital mandibular hypoplasia were finally included in the final analyses. In the healthy control cohorts with available facial bone or head CT scans (n = 518), 42 cases were included after demographically matching the controls to 42 patients with mandibular hypoplasia. The standardized difference of age, sex, height, and weight were 0.0067, − 0.0905, − 0.01291, and 0.1425, respectively.

Among the patients with mandibular hypoplasia, the diagnosis was PRS (n = 5, 11.9%), TCS (10, 23.8%), HM (15, 35.7%), and GS (12, 28.6%). In the total study cohort, ten patients with difficult airway were revealed after the review of electronic medical records. These individuals consisted of four patients with PRS, two with TCS, two with HM, and two with GS. Seven patients were ≤ 5 years old, and three patients were > 5 years old. One patient presented difficult mask ventilation (2.4%), and eight presented difficult laryngoscopy or intubation (19.0%); one patient had both (2.4%). Thirty-nine patients underwent 41 surgery under general anesthesia. For airway maintenance during the induction, direct laryngoscopy was used in 35 (85.4%) cases, a supraglottic airway was used in two (4.9%), a videoscope was used in two (4.9%), a bronchoscope was used in one (2.4%), and tracheostomy was used in one (2.4%). Eight patients (19.0%) received tracheostomy during the perioperative period. In these patients, 4 patients received planned elective tracheostomy in the operating room under general anesthesia, and they had difficult airway; one patient with difficult mask ventilation and failed intubation, and three patients with difficult intubation. Four patients received tracheostomy under local anesthesia with sedation in the intensive care unit before surgery. Two patients underwent planned elective tracheostomy for prolonged mechanical ventilation after birth in the intensive care unit, but they did not have a difficult airway. Two patients underwent emergent tracheostomy after failed intubation in the intensive care unit because of life-threatening airway compromise. Consequently, six patients with receiving tracheostomy were included in a difficult airway group.

**Figure 2:**
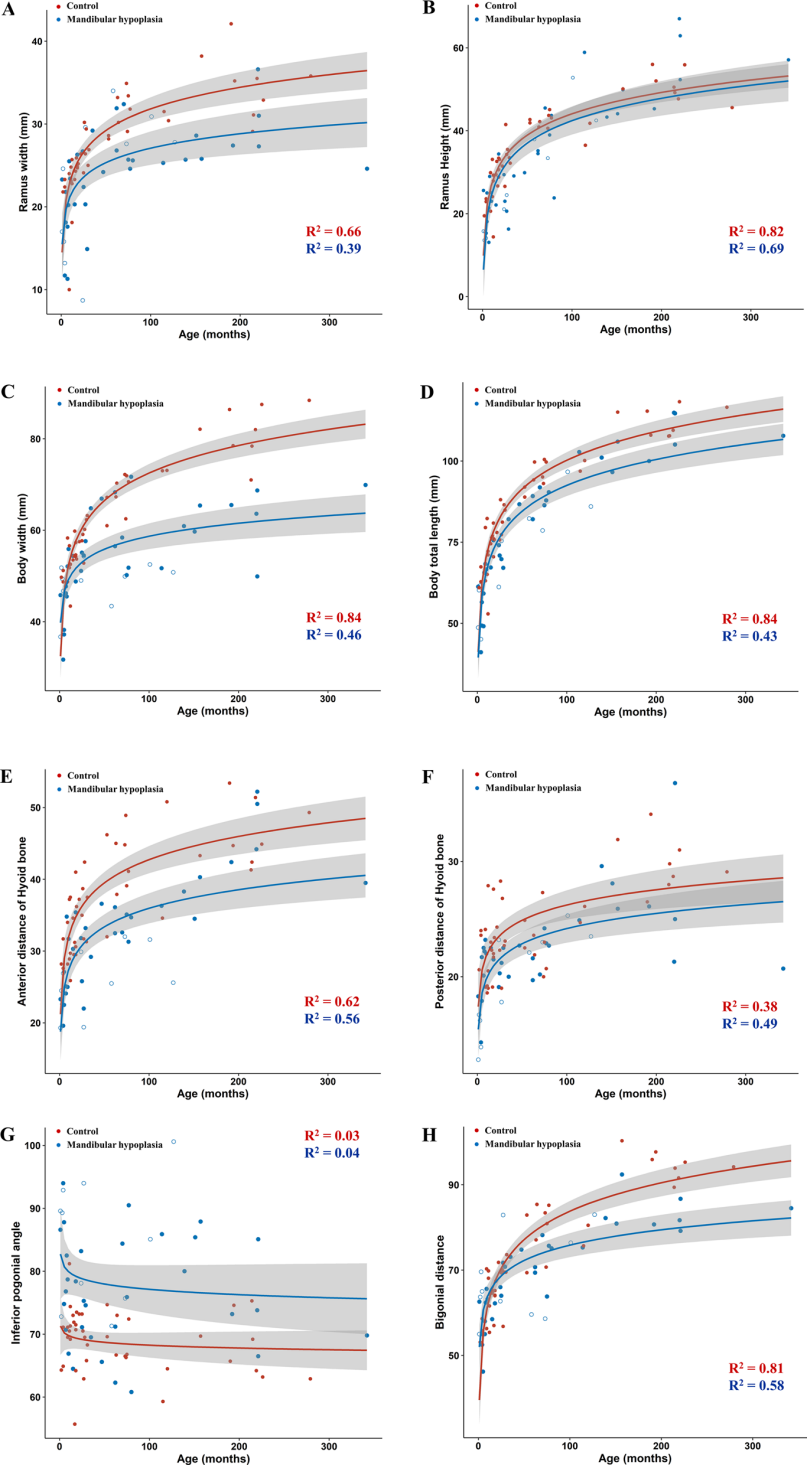
Developmental differences in ramus width and height (**A**, **B**), body width and the total length of the mandible (**C**, **D**), anterior and posterior distance of hyoid bone (**E**, **F**), inferior pogonial angle, and bigonial distance (**G**, **H**) according to age between the mandibular hypoplasia and the control groups. The red line indicates the developmental curve of the healthy controls. The blue line indicates the developmental curve of the patients with mandibular hypoplasia. The grey zone indicates a 95% confidence interval. The open circle represents patients with a difficult airway among those with mandibular hypoplasia.

There were significant discrepancies in tongue position relative to the anterior nasal spine and palate between patients with mandibular hypoplasia and healthy controls (10.8 ± 4.2 mm vs. 8.3 ± 4.1 mm, *P* = 0.007 and 3.1 (1.4–6.1) mm vs. 5.2 (3.5–7.3) mm, *P* = 0.017; Table 1). Anterior distance and posterior distance of the hyoid bone (HAD and HPD) were shorter in patients with mandibular hypoplasia than in healthy controls [31.6 (25.1–35.4) mm vs. 37.4 (31.3–43.3) mm, *P* < 0.001 and 21.8 ± 4.2 mm vs. 24.2 ± 3.9 mm, *P* = 0.009]. On 3D CT measurements, body total length, body width, and ramus width were significantly lesser in patients with mandibular hypoplasia, and gonial and inferior pogonial angles (IPA) were greater. The other parameters did not differ between the two groups. Ramus width and height, body width, the total length HAD and HPD, and bigonial distance in both groups were significantly related to age in the linear regression with logarithmic transformations (Fig. 2).

**Table 1:** Clinical characteristics and three-dimensional computed tomographic measures of the upper airway between the mandibular hypoplasia and control groups.

	Mandibular hypoplasia (N = 42)	Control (N = 42)	*P* value
Age at CT^a^ scan (months)	32.0 (8.0–101.0)	27.5 (15.0–77.0)	0.758
Sex (male, %)	17 (40.5)	20 (47.6)	0.660
Height (cm)	94.1 (72.0–117.5)	84.7 (77.0–120.1)	0.681
Weight (kg)	13.2 (9.3–23.1)	13.2 (10.6–23.4)	0.558
**Airway volume (cm**^**3**^**)**			
Nasopharynx	0.32 (0.06–0.75)	0.25 (0.75–0.49)	0.875
Oropharynx	1.74 (1.05–3.15)	2.59 (1.32–4.24)	0.078
Hypopharynx	1.31 (0.45–2.27)	1.37 (0.70–2.55)	0.462
Oral cavity	2.35 (0.66–4.59)	2.94 (1.86–5.99)	0.294
**Airway area (cm**^**2**^**)**			
Nasopharynx	3.78 (1.26–7.01)	3.04 (1.49–6.26)	0.988
Oropharynx	11.66 (8.17–18.47)	15.04 (9.50–21.23)	0.053
Hypopharynx	9.46 (6.14–15.35)	11.34 (7.27–15.22)	0.361
Oral cavity	20.01 (6.08–22.81)	20.23 (12.81–27.29)	0.487
**Tongue**			
Length (mm)	74.1 (62.7–86.3)	67.3 (58.5–83.8)	0.413
Height (mm)	39.1 (32.1–45.0)	38.2 (32.0–42.6)	0.642
Area (cm^2^)	11.68 (7.93–15.01)	10.30 (8.10–15.80)	0.778
Relative position to ANS^b^ (mm)	10.8 ± 4.2	8.3 ± 4.1	0.007
Relative position to Palate (mm)	3.1 (1.4–6.1)	5.2 (3.5–7.3)	0.017
**Hyoid (mm)**			
Hyoid anterior distance	31.6 (25.1–35.4)	37.4 (31.3–43.3)	< 0.001
Hyoid posterior distance	21.8 ± 4.2	24.2 ± 3.9	0.009
Craniocaudal length	33.5 (28.6–41.0)	32.8 (29.2–37.9)	0.950
Mandibular plane distance	5.6 ± 4.3	5.3 ± 5.5	0.825
**Mandible**			
Ramus height (mm)	32.7 ± 14.7	36.2 ± 10.2	0.211
Ramus width (mm)	23.9 ± 6.3	27.8 ± 5.8	0.004
Body total length (mm)	78.2 ± 19.7	86.6 ± 17.0	< 0.001
Body width (mm)	53.6 ± 9.4	63.3 ± 11.8	0.038
Gonial angle (°)	136.2 ± 6.4	129.1 ± 5.4	< 0.001
Inferior pogonial angle (°)	78.6 ± 9.6	69.0 ± 4.8	< 0.001
Bigonial distance (mm)	69.4 ± 10.7	73.6 ± 13.3	0.114

Values are mean ± (standard deviation) or median (interquartile range).

^a^*CT* Computed tomography.

^b^*ANS* Anterior nasal spine.

The area and volume of the oropharynx, ramus height of mandible, and HPD were significantly lesser in patients with mandibular hypoplasia in the ≤ 5 year-old group only (Table 2). Tongue position relative to the anterior nasal spine, HAD, ramus and body width, body total length, gonial angle, and inferior pogonial angle were significantly different between the groups, regardless of age.

**Table 2:** Clinical characteristics and three-dimensional computed tomographic measures of upper airway between the mandibular hypoplasia and control groups, stratified by stage of dentition.

	≤ 5 years old group			> 5 years old group		
	Mandibular hypoplasia (n = 24)	Control (n = 26)	*P* value	Mandibular hypoplasia (n = 18)	Control (n = 16)	*P* value
Age at CT^a^ scan (months)	9.5 (4.5–26.0)	17.5 (11.0–26.0)	0.203	120.5 (75.0–192.0)	138.5 (74.5–214.5)	0.717
Sex (male, %)	9 (50.0)	8 (50.0)	1.000	8 (33.3)	12 (46.2)	0.525
Height (cm)	73.5 ± 16.0	78.6 ± 13.1	0.252	130.1 ± 22.9	140.0 ± 28.8	0.304
Weight (kg)	10.0 (5.7–12.1)	10.6 (9.1–11.7)	0.221	25.4 (19.2–44.2)	39.0 (23.1–58.1)	0.138
**Airway volume (cm**^**3**^**)**						
Nasopharynx	1.00 (0.02–0.29)	0.16 (0.06–0.42)	0.291	0.91 (0.52–1.78)	0.52 (0.24–1.57)	0.518
Oropharynx	1.18 (0.30–1.56)	1.43 (1.03–2.57)	0.022	3.05 (2.57–4.46)	4.65 (3.29–6.72)	0.075
Hypopharynx	0.53 (0.26–0.95)	0.85 (0.41–1.30)	0.097	2.61 (1.82–5.35)	4.08 (2.17–5.89)	0.443
Oral cavity	2.08 (0.55–4.79)	2.96 (1.99–4.63)	0.448	2.35 (1.37–4.59)	2.91 (1.86–7.34)	0.481
**Airway area (cm**^**2**^**)**						
Nasopharynx	1.74 (0.65–3.78)	2.45 (1.36–4.01)	0.289	7.66 (5.26–1.02)	7.17 (3.54–11.18)	0.799
Oropharynx	8.42 (4.02–10.80)	10.90 (8.57–13.88)	0.012	19.43 (12.92–21.64)	21.66 (18.21–27.74)	0.064
Hypopharynx	6.21 (3.32–8.14)	8.12 (5.17–11.27)	0.075	16.15 (12.08–26.60)	22.50 (14.43–29.22)	0.330
Oral cavity	17.31 (4.57–25.47)	29.63 (19.85–46.33)	0.579	22.65 (11.78–31.35)	31.42 (11.35–44.60)	0.541
**Tongue**						
Length (mm)	67.0 ± 11.4	61.6 ± 9.3	0.075	86.5 ± 15.0	61.6 ± 9.3	0.199
Height (mm)	36.4 ± 7.2	32.9 ± 6.0	0.064	44.5 ± 9.7	32.9 ± 6.0	0.080
Area (cm^2^)	8.94 ± 2.53	8.70 ± 2.09	0.714	17.37 ± 5.08	19.10 ± 5.87	0.364
Relative position to ANS^b^ (mm)	11.4 ± 4.4	9.0 ± 3.7	0.045	10.0 ± 3.8	7.1 ± 4.4	0.047
Relative position to Palate (mm)	4.3 ± 3.8	5.6 ± 3.3	0.197	3.9 ± 3.9	5.3 ± 2.8	0.259
**Hyoid bone (mm)**						
Hyoid anterior distance	26.9 ± 5.1	33.6 ± 5.0	< 0.001	37.2 ± 6.8	44.6 ± 5.2	0.001
Hyoid posterior distance	19.8 ± 3.1	22.7 ± 2.9	0.001	24.5 ± 4.1	26.6 ± 4.1	0.140
Craniocaudal length	29.7 ± 7.8	29.7 ± 4.6	0.977	41.1 ± 8.7	45.3 ± 11.5	0.230
Mandibular plane distance	5.1 (2.5–6.9)	3.4 (1.4–6.1)	0.125	4.5 (2.4–8.8)	6.5 (0.9–11.8)	0.918
**Mandible**						
Ramus height (mm)	22.6 ± 6.7	30.0 ± 6.7	< 0.001	46.2 ± 11.1	46.3 ± 5.6	0.966
Ramus width (mm)	20.8 ± 6.3	24.4 ± 3.9	0.023	28.1 ± 3.3	33.4 ± 3.5	< 0.001
Body total length (mm)	64.4 ± 12.0	75.4 ± 9.4	0.001	96.6 ± 10.7	104.9 ± 8.5	0.019
Body width (mm)	49.4 ± 8.4	55.5 ± 5.2	0.004	59.2 ± 7.8	76.0 ± 7.6	< 0.001
Gonial angle (°)	135.3 ± 6.9	131.3 ± 4.8	0.021	137.4 ± 5.6	125.4 ± 4.2	< 0.001
Inferior pogonial angle (°)	78.7 ± 9.2	69.8 ± 4.8	< 0.001	78.6 ± 10.5	67.5 ± 4.5	< 0.001
Bigonial distance (mm)	63.3 ± 8.0	65.1 ± 6.9	0.396	77.5 ± 8.1	87.3 ± 8.8	0.002

Values are mean ± (standard deviation) or median (interquartile range).

^a^*CT* Computed tomography.

^b^*ANS* Anterior nasal spine.

**Figure 3:**
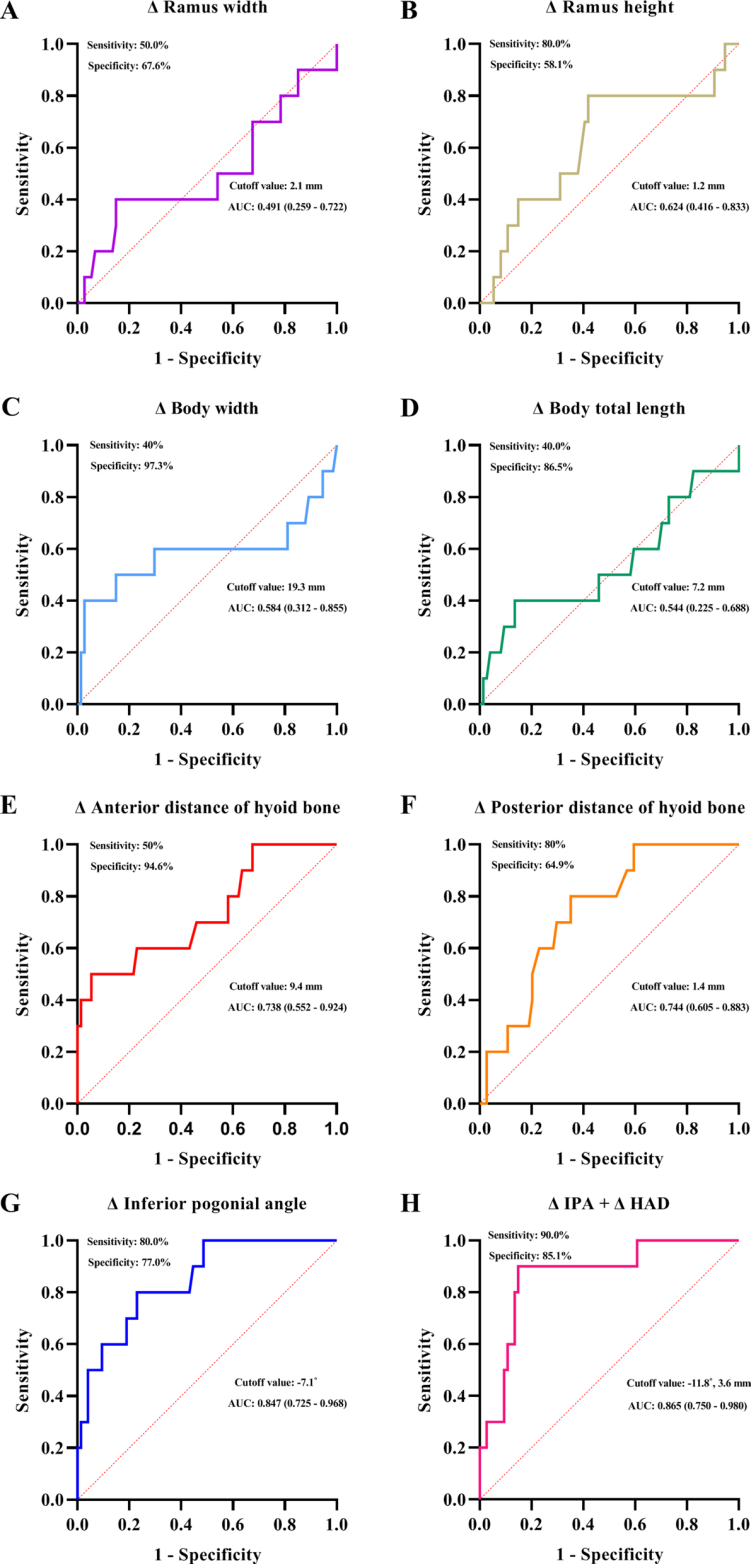
ROC curves for prediction of a difficult airway using ∆ramus width and height (**A**, **B**), ∆body width and the total length of the mandible (**C**, **D**), ∆anterior and posterior distance of hyoid bone (**E**, **F**), ∆ inferior pogonial angle (IPA), and ∆IPA plus ∆anterior distance of hyoid bone (HAD) (**G**, **H**). ∆ indicates the differences between estimated value according to the regression equation and measurement value of each parameter.

HAD, HPD, body total length, body width, and IPA of mandible were significantly different in patients with difficult airway (n = 10) compared to those with non-difficult airway (n = 74). After regression analysis using these parameters, HAD, HPD, and IPA were associated with a difficult airway [HAD: odds ratio, 0.83, 95% confidence interval (CI), (0.71–0.96), *P* = 0.01; HPD: 0.78 (0.62–0.98), *P* = 0.04; IPA: 1.11 (1.01–1.22), *P* = 0.02; Table 3]. After adjustment for age and sex, HAD and IPA remained significant [0.79 (0.64–0.97), *P* = 0.03 and 1.10 (1.01–1.21), *P* = 0.04]. From the ROC curve analysis (Fig. 3), the area under the curve (AUC) of ∆IPA (0.847, 95% CI: 0.725–0.968) was the greatest among the single parameters and the cutoff point of ∆IPA was − 7.1° (sensitivity 80.0%, specificity 77.0%; Fig. 3G). The addition of ∆HAD to ∆IPA improved the AUC, sensitivity, and specificity. (AUC: 0.865, 95% CI: 0.751–0.980, sensitivity: 90.0%, specificity: 85.1%, Fig. 3H).

**Table 3:** Crude, and age and sex-adjusted logistic regression for the prediction of difficult airway.

Variables	Crude		Age and sex-adjusted	
	OR (95% CI)^a^	*P* value	OR (95% CI)	*P* value
Hyoid anterior distance	0.83 (0.71–0.96)	0.01	0.79 (0.64–0.97)	0.03
Hyoid posterior distance	0.78 (0.62–0.98)	0.04	0.78 (0.60–1.02)	0.07
Ramus height	0.96 (0.91–1.02)	0.17	0.97 (0.86–1.10)	0.65
Ramus width	0.97 (0.87–1.09)	0.64	1.02 (0.88–1.18)	0.79
Body total length	0.97 (0.93–1.01)	0.11	0.97 (0.89–1.05)	0.45
Body width	0.90 (0.82–1.00)	0.05	0.93 (0.82–1.05)	0.22
Inferior pogonial angle	1.11 (1.01–1.22)	0.02	1.10 (1.01–1.21)	0.04
Bigonial distance	0.97 (0.91–1.04)	0.46	1.03 (0.92–1.14)	0.61

^a^Odds ratios (OR) and 95% confidence intervals (CI).

**Figure 4:**
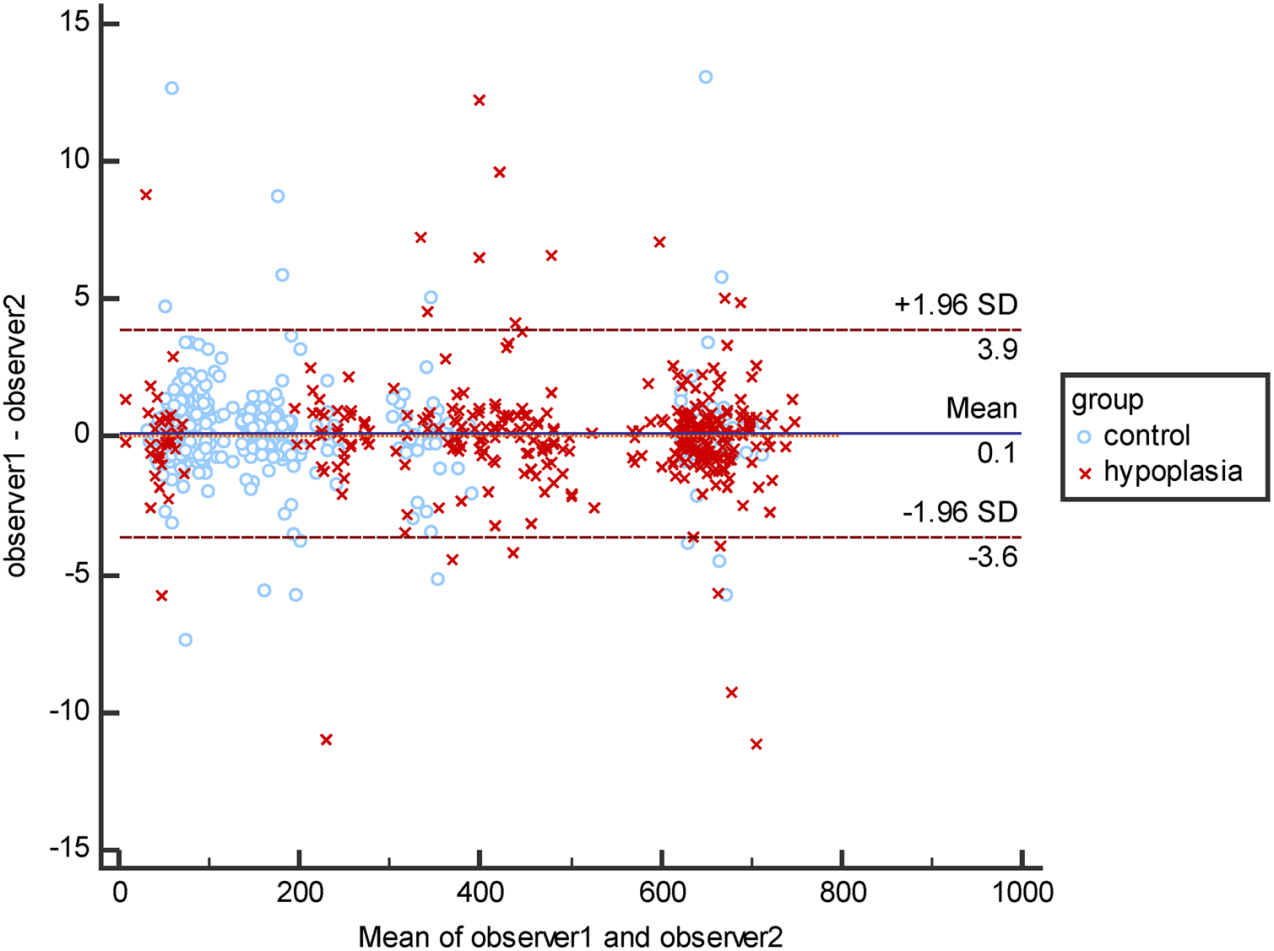
Bland–Altman plot for evaluating inter-rater reliability.

Inter-rater reliability was measured by landmarks on 40 CT scans and was evaluated using the Bland–Altman plot on the Euclid distance between landmarks and origins (Fig. 4). The mean ± SD of landmark errors in the hypoplasia and the controls by observer 1 and observer 2 were 2.19 ± 2.50 mm and 1.81 ± 1.18 mm, respectively.

## Discussion

This study produced three main findings. First, patients with mandibular hypoplasia showed different growth patterns in upper airway anatomical structures when compared to healthy controls. We found significant between-group differences in the relative tongue position, hyoid distance, and mandible measurements, but not in the tongue size or airway volume. Second, we related cephalometric parameters associated with anatomic development retardation and their outcomes, which implicated an airway management strategy for children with mandibular hypoplasia. Third, we suggested IPA and HAD as clinically meaningful risk factors for airway failure in children with mandibular hypoplasia. Clinically, these measures may be useful predictors of difficult intubation because HAD can be easily estimated by measuring the thyromental distance.

To our knowledge, this is the first and largest cohort study to provide digitalized quantitative measurement of 30 anatomical landmarks of 84 3D CT scan datasets for current disease entities. Our results showed significant between-group and age-related differences in most measurement, but some features were not. The tongue position was relatively away from the anterior nasal spine and relatively closer to the palate in patients with mandibular hypoplasia. This suggests that taller tongue height and more posterior tongue position in these individuals, consistent with a diagnosis of glossoptosis^15^. Glossoptosis is one of the clinical triad in patients with PRS and has been associated with oropharyngeal obstruction^16^. Although there were relatively few patients with PRS in the cohort of patients with mandibular hypoplasia (11.9%), glossoptosis was one of the prominent morphological features of mandibular hypoplasia. It is possible that the tongue is consequently be shifted posteriorly as a smaller mandible has less-anterior projection^17^. HAD and HPD describe the external (mandible) and internal (hyoid) bony relationships that influence oral and pharyngeal soft tissue shape^15^. In this study, they were significantly shorter in patients with mandibular hypoplasia. This suggested a compressed hyoid position, which could easily narrow the compliant airway^15^. A relatively long distance between the mandibular plane and the hyoid bone caused by a short mandibular ramus is traditionally considered a risk factor for a difficult airway^2,18,19^. However, we did not find significant between-group differences for this factor in our study. This inconsistency may be explained, at least partially, by no differences in the mandibular rami in older children.

Most 3D CT measurements of the mandible (ramus width, total body length, body width) in the mandibular hypoplasia group were significantly shorter than the same measures in healthy controls. This indicated micrognathia, the characteristic of shorter mandibles. Currently, the diagnosis of micrognathia is largely subjective, and the objective parameters are not present^20^. Reference guides for normal mandibular dimensions within various age, sex, and racial groups are also needed for clinical application. This study provides objective parameters of micrognathia and normal mandibular dimensions according to age in Asian children, contributing to determine diagnostic criteria for micrognathia. Micrognathia induces posterior regression of the tongue and a small hyoid-mental space, potentially resulting in hypopharyngeal collapse^2,16^. It can also cause narrowing of the submandibular space, which can obstruct glottic visualization and produce a difficult airway during laryngoscopy because of an inability to accommodate the displaced tongue^21^. In patients with mandibular hypoplasia, airway obstruction develops at various levels—from oropharyngeal obstruction due to glossoptosis and compressed hyoid position to hypopharyngeal obstruction due to micrognathia. Additionally, perioperative difficult airway is also common in this cohort^16^.

In pediatric airway management, measurements of surface landmarks are guidelines for determining the proper size of the nasopharyngeal or oropharyngeal airway according to age and sex^22,23^. These nomograms are important for understanding normal development patterns and the relative structural relationships of the pediatric airway. As a series study of those, current study determined growth patterns of upper airway structures between children with mandibular hypoplasia and healthy controls. Measurements of upper airway structures were well correlated with age between the groups. Intriguingly, we observed increased between-group measures of per child growth, the differences of ramus width, body width, and the gonial angle, but not ramus height. One explanation could be a unique growth pattern for ramus height. Increases in ramus height are especially accelerated after the first three years of life, peaking at age five or six^24^. This accelerating growth pattern seemed to be especially present in children (> 5 years) with mandibular hypoplasia. Consequently, among older children, ramus height was similar, although there were significant between-group differences in all other mandibular measurements.

Among patients with mandibular hypoplasia, eight (19.0%) underwent tracheostomy during the perioperative period. This is consistent with previous studies which reported that 18–23% in patients with PRS and TCS received tracheostomy^25,26^. To maintain airway patency, four patients (10.3%) received tracheostomy intraoperatively out of the ten patients (25.6%) with difficult airways. In the prior reports^27,28^, a difficult airway was present in 5.8–6.0% of cases and was mostly managed effectively in healthy patients undergoing elective surgery. The incidence of difficult mask ventilation combined with difficult tracheal intubation was 0.4%^29^. Therefore, our results indicated that mandibular hypoplasia was significantly associated with a difficult airway, and airway management in these patients was challenging compared to that in healthy controls.

IPA and HAD were significantly associated with a difficult airway after adjusting for age and sex. IPA was defined as the angle formed by the gonion to the pogonion to the gonion, and corresponded to the triangular area of the horizontal plane of the mandible. This angle was significantly wider in patients with a difficult airway compared to those with a non-difficult airway. However, the bigonial distance—which represented the length of the base of this triangular area—did not differ between the groups, suggesting that the mandible horizontal dimension was significantly less in patients with difficult airways. This might lead to a narrowed submandibular space. As mentioned above, a short HAD could result in a narrowed compliant airway. Therefore, a wide IPA and short HAD in patients with mandibular hypoplasia could induce significant narrowing of the submandibular space, resulting in a difficult airway. Interestingly, these parameters were also associated with tracheostomy in patients with PRS, which is consistent with our results^15^. We also calculated the differences between the estimated normal value according to the age and measurement value of each parameter. After ROC curve analysis using the differences, ∆IPA and ∆HAD could screen the difficult airway in patients with mandibular hypoplasia. Given our results, we believe that IPA and HAD may be risk factors for a difficult airway in patients with mandibular hypoplasia. Especially, assessment of HAD seemed to be similar to that of hyoid-mental distance, which is an important surrogate for predicting a difficult airway^30^. Hence, the preoperative hyoid-mental distance may aid in screening patients with difficult airways in those with mandibular hypoplasia if the digitalized quantitative measurements are not available in individual clinical settings.

Radiologic assessment using CT, magnetic resonance imaging, X-ray, and ultrasound display the anatomical features of the upper airways well and are recommended for evaluation of difficult airway^31,32^. The 3D CT images can provide detailed imaging of upper airway structures, including bony and soft tissues, and quantify the tongue position and mandibular configuration, thereby identifying patients with mandibular hypoplasia. Many of these patients have considered surgical treatment, and facial CT images are almost needed. Therefore, it may be feasible to use the 3D analysis to evaluate the airway and make an airway management plan.

Our study had certain limitations related to the methodology. First, we did not include additional information related to airway assessment—such as the Cormack–Lehane classification which are clinical difficult airway predictors—because of the retrospective nature of our data collection process. Second, the small sample size limited the power of our study and the robustness of our conclusions. Relative to the published literature, however, our study included the largest group of patients with mandibular hypoplasia supported by objective airway morphology data. Additionally, this was the first study to compare the airways and facial skeletal morphologies in children with those in a healthy control group. Although prospective validation is needed, we believe this study represents the first step toward the development of an objective parameters-based decision tool for airway management in patients with mandibular hypoplasia. In previous our study, we developed the patient-specific and hyper-realistic phantom for difficult intubation simulation using 3D printing^33^. To enrich our understanding the association between specific parameters and difficult airway in children with mandibular hypoplasia, we plan to examine the validity of the association via a manikin airway simulation model.

## Conclusion

This study attempted to understand the growth dynamics of the tongue, airway, and mandible in children with mandibular hypoplasia. Digitalized quantitative measurements of upper airway structures revealed significant age-related differences in relative tongue position, hyoid distance, and mandible measures between children with mandibular hypoplasia and healthy controls. Mandibular hypoplasia was significantly associated with a difficult airway and these patients required attentive airway management. IPA and HAD may be clinically meaningful risk factors for predicting a difficult airway in patients with mandibular hypoplasia. This information can assist our understanding of the airways in children with mandibular hypoplasia and allow us to establish proper airway management protocols in clinical practice.

## Supplementary information

Supplementary information

## Data Availability

The datasets generated during and/or analysed during the current study are available from the corresponding author on reasonable request.
