# Supplementary material for: Developmental features and predicting airway failure risk in critically ill children with mandibular hypoplasia using 3D computational tomographic analysis
Source: Sci Rep. 2021 May 10;11:9881. doi: 10.1038/s41598-021-89302-4 (PMC8110979; doi:10.1038/s41598-021-89302-4)
Supplement: Supplementary file 1 — Supplementary information [file 41598_2021_89302_MOESM1_ESM.pdf]

Developmental features and predicting airway failure risk in critically ill children with mandibular hypoplasia using 3D computational tomographic analysis

Doo-Hwan Kim,[1†] Eunseo Gwon,[2†] Junheok Ock,[2] Jong-Woo Choi,[3] Jee Ho Lee,[4] Sung-Hoon Kim,[1*] and Namkug Kim[2*]

[1]Department of Anesthesiology and Pain Medicine, Asan Medical Center, University of Ulsan College of Medicine, Seoul, Korea. [2]Department of Convergence Medicine, Asan Medical Center, University of Ulsan College of Medicine, Seoul, Korea. [3]Department of Plastic surgery, Asan Medical Center, University of Ulsan College of Medicine, Seoul, Korea. [4]Department of Oral and Maxillofacial Surgery, Asan Medical Center, University of Ulsan College of Medicine, Seoul, Korea.

Supplementary Tables

Table S1. Definitions of the important cephalometric landmarks for 3D analysis

Landmark	Abbreviation	Definition
**Cranium**		
Orbitale *	Or	The most inferior point on the lower rim of the orbit
Porion *	Po	The highest superior point on the upper rim of the external auditory meatus
Nasion **	Na	The junction between the nasal and frontonasal sutures
Opisthion **	Op	The most posterior point on the posterior margin of the foramen magnum
Posterior aspect of vomer ***	Pa_V	The junction between posterior aspect border of the vomer and sphenoid
Basion	Ba	The most anterior border of the foramen magnum
**Maxilla**		
Anterior Nasal Spine	ANS	The most anterior point of the anterior nasal spine of the maxilla
**Mandible**		
B-point	B	The most concave point on mandibular symphysis
Superior of condylion	S_Co	The most superior point of the mandibular condyle head
Posterior of condylion	P_Co	The most posterior point of the mandibular condyle head
Anterior inner aspect of ramus	Aia_R	The most concave anterior point of inner aspect of ramus of mandible
Posterior inner aspect of ramus	Pia_R	The most concave posterior point of ramus of mandible
Gonion	Go	The most inferior, posterior and lateral point of the mandibular angle
Pogonion	Pog	The most anterior point of the bony chin
Menton	Me	The most inferior point on mandibular symphysis

**Cervical vertebrae**		
Anterior Inferior aspect of C2	AIa_C2	The most anterior and inferior border of the cervical vertebrae C2
Anterior Inferior aspect of C4	AIa_C4	The most anterior and inferior border of the cervical vertebrae C4
Anterior aspect of C2	Aa_C2	The most anterior rim of the cervical vertebrae C2
Anterior aspect of C3	Aa_C3	The most anterior rim of the cervical vertebrae C3
**Larynx**		
Center of hyoid bone	H	The superior middle point of the hyoid body
Inferior aspect of vallecula	Ia_V	The inferior aspect point of epiglottic vallecula in midsagittal plane
Anterior aspect of tongue	Aa_T	The most anterior border of the tongue
Superior aspect of tongue	Sa_T	The most superior rim of the tongue

* Landmarks for defining the average Frankfort horizontal plane, ** Landmarks for defining the midsagittal plane, *** Landmarks for defining the coronal vomer plane.

Table S2. Definitions of the additional cephalometric measurements for 3D analysis

Measurement	Definition
Borders of the Airway Divisions	
Nasopharynx	Anterior: Coronal vomer plane<br>Inferior: Plane parallel to the average Frankfort horizontal plane passing through the anterior nasal spine (AFH_p//ANS)<br>Posterior: Pharyngeal wall
Oropharynx	Anterior: Coronal vomer plane<br>Superior: AFH_p//ANS<br>Inferior: Plane parallel to AFH_p passing through AIa_C2 (AFH_p//AIa_C2)<br>Posterior: Pharyngeal wall
Hypopharynx	Anterior: Coronal vomer plane<br>Superior: AFH_p//AIa_C2<br>Inferior: Plane parallel to AFH_p passing through AIa_C4 (AFH_p//AIa_C4)<br>Posterior: Pharyngeal wall
Airway Divisions	
Nasopharynx_volume (mm^3)	Volume measurement of the 3D part of nasopharynx according to the Borders of the Airway Divisions
Nasopharynx_area (mm^2)	Area measurement of the 3D part of nasopharynx according to the Borders of the Airway Divisions
Oropharynx_volume (mm^3)	Volume measurement of the 3D part of oropharynx according to the Borders of the Airway Divisions
Oropharynx_area (mm^2)	Area measurement of the 3D part of oropharynx according to the Borders of the Airway Divisions

Hypopharynx_volume (mm^3)	Volume measurement of the 3D part of hypopharynx according to the Borders of the Airway Divisions
Hypopharynx_area (mm^2)	Area measurement of the 3D part of hypopharynx according to the Borders of the Airway Divisions
Tongue size and position	
Tongue length (mm)	The curvilinear distance between 2 points in the 2D-midsagittal plane: The inferior aspect of the vallecula and anterior aspect of the tongue
Tongue height (mm)	The distance between 2 points: The inferior aspect of the vallecula and superior aspect of the tongue
Tongue area (mm^2)	The area contained within an outline along the superior surface of the tongue in the 2D-midsagittal plane: Passing through the inferior aspect of the vallecula, center of the hyoid bone, and posterior aspect of the mandible
Position relative to anterior nasal spine (mm)	The distance from the plane perpendicular to AFH_p through the anterior aspect of the tongue to the plane perpendicular to AFH_p through the ANS
Position relative to plate (mm)	The distance from the plane parallel to AFH_p passing through the ANS to the superior aspect of the tongue
Hyoid position (mm)	
Hyoid anterior distance	The distance between 2 points: Center of the hyoid bone and pogonion
Hyoid posterior distance	The perpendicular distance from the center of the hyoid bone to a line connecting the anterior aspects of C2 and C3
Craniocaudal position	The perpendicular distance from the center of the hyoid bone to AFH_p//ANS
Mandibular plane distance	The distance from the mandibular plane to the hyoid bone
Mandible measures	
Ramus height (mm)	The distance from the condylion superior to the gonion
Ramus width (mm)	The distance between 2 points: Anterior inner aspect of the ramus and posterior inner aspect of the ramus
Body total length (mm)	The distance between 2 points: Pogonion and superior condylion

Body width (mm)	The distance between 2 points: B-point and gonion
Gonial angle (°)	The angle between 3 points: Menton, gonion, and posterior condylion
Inferior pogonial angle (°)	The angle between the 3 points: Pogonion and bilateral gonion
Bigonial distance (mm)	The distance between bilateral gonion
Other	
Oral cavity volume (mm^3)	Volume measurement of the 3D part of the oral cavity
Oral cavity area (mm^2)	Area measurement of the 3D part of the oral cavity
